# IGHG1 Regulates Prostate Cancer Growth via the MEK/ERK/c-Myc Pathway

**DOI:** 10.1155/2019/7201562

**Published:** 2019-07-04

**Authors:** Jing Chu, Yutong Li, Zhihai Deng, Zhenlin Zhang, Qun Xie, Heyuan Zhang, Weifeng Zhong, Bin Pan

**Affiliations:** ^1^Department of Urology, Zhuhai People's Hospital, Zhuhai, 519000, China; ^2^Department of Urology, The First Affiliated Hospital of Jinan University, Guangzhou, 510630, China; ^3^Department of Urology, Gaozhou People's Hospital, Gaozhou, 525200, China; ^4^Department of Urology, Meizhou People's Hospital, Meizhou, 514031, China

## Abstract

Increasing evidence indicates that immunoglobulins are important for the regulation of various cancers including prostate cancer (PCa). However, the underlying mechanisms of IgG regulated PCa development remain to be further explored. Here, we demonstrated that IgG1 heavy chain (IGHG1) was increased in tissues from PCa patients. Inhibition of IGHG1 by antibody blocking or genetic knockdown suppressed cell growth and induced cell cycle arrest and ultimate apoptosis. Expression levels of c-Myc were positively correlated with the levels of IGHG1. Furthermore, MEK/ERK/c-Myc pathway lied downstream of IGHG1 in cultured prostate cancer cells. Inhibition of IGHG1 restrained the tumor growth in nude mice and inactivated MEK/ERK/c-Myc pathway both* in vitro* and* in vivo*. These findings suggest that IGHG1 play a crucial role during the development of prostate cancer and inhibition of IGHG1 may be a potential therapy in the treatment of PCa.

## 1. Introduction

Prostate cancer (PCa), the second leading cause of cancer-related death of man, is one of the most common cancers of urinary system [1]. Same as other cancers, metastasis induces the morbidity and mortality of PCa patients [2, 3]. Hormone therapy is one the most common treatments of PCa. Restricted androgen level shrinks cancer volume and delays the development of tumors. However, hormone therapy only results in a medium survival time of around 12 months in patients with metastatic PCa [4]. Thus, further elucidation of PCa development of molecular mechanisms and exploration of new therapeutic targets and reliable biomarkers for detection of metastatic potential are of specific importance.

It is commonly known that immunoglobulins (IgG) are produced only by B lymphocytes and plasma cells; however, many nonlymphoid cells are reported to produce IgG, especially in cancer cells, such as breast cancer cells [5–7], colorectal cancer cells [8, 9], papillary thyroid cancer cells [10], and prostate cancer cells [11, 12]. IgG secreted by human cancers is reported to promote cancer cell proliferation* in vitro* [5]. IgG expressed in a variety of neoplasms shows correlation with proliferation markers and tumor grades [5]. Moreover, genetic knockdown of IgG by siRNA approaches inhibits cancer cell proliferation* in vitro *and* in vivo* [13]. The role of IgG in prostate cancer remains obscure. Our previous reports showed that IgG1 heavy chain (IGHG1) was expressed in LNCaP and PC3 prostate cancer cell lines, and inhibition of IGHG1 suppressed cell viability of PCa cells. However, the regulatory mechanisms of IGHG1 regulated PCa development remain to be further explored.

In this study, we further determined the effect of IGHG1 and investigated the cellular mechanism of IGHG1 in prostate cancer. We found that IGHG1 was upregulated in clinical prostate cancer tissue from PCa patients and downregulation of IGHG1 reduced the growth and proliferation of PCa cells. Further, the expressions of IGHG1 and c-Myc were positively correlated in PCa samples. Inhibition of IGHG1 suppressed the activation of MEK/ERK/c-Myc pathway* in vitro* and* in vivo*.

## 2. Methods and Materials

### 2.1. Ethics Statement

All human experiments were approved by the Jinan University and in accordance with the Declaration of Helsinki. Informed consent was received from all participating subjects prior to the study. 164 cases of human tissue samples of prostate cancer and 55 cases of benign prostatic hyperplasia were obtained from patients in the First Affiliated Hospital of Jinan University, from June 2010 to June 2014. All animal procedures followed the humane care guidelines of the Chinese National Institute of Health, and the protocols were approved by the Committee on Animal Research of Jinan University.

### 2.2. Cell Culture

Cell lines human prostate cancer DU145 and PC3 were from the First Affiliated Hospital of Jinan University. Cells were maintained in medium RPMI 1640 (HyClone, Logan, UT, USA) supplemented with 10% fetal bovine serum (FBS, Sigma-Aldrich-Chemie, Steinheim, Germany), penicillin (Sigma, 100 U/ml), and streptomycin (Sigma, 100 *μ*g/ml) at 37°C in a humidified atmosphere with 5% CO_2_, as previously reported [12].

### 2.3. siRNA and Transfection

siRNA experiments were performed as previously reported [12]. Briefly, when cells reached 40% confluence, the siRNA fragments were transfected by Lipofectamine RNAiMax (Invitrogen, Grand Island, NY, USA) accordingly. The transfection efficiency was determined by western blot. Scrambled siRNA sequence was used as control (si-Ctrl), comparing with experiment group (si-IGHG1).

### 2.4. Western Blot

Cells were lysed in sample buffer and subjected to SDS-polyacrylamide gel electrophoresis as described previously [14]. Primary antibodies against IGHG1, MEK, phosphorylated-MEK (p-MEK), ERK, phosphorylated-ERK (p-ERK), c-Myc, p21, and Cyclin D1 were obtained from Santa Cruz Biotechnology, Santa Cruz, CA, USA. GAPDH (Santa Cruz) was used as the loading control. Polyvinylidene fluoride (PVDF) membranes (Millipore, Boston, MA, USA) transferred with proteins were washed and incubated with the appropriate horseradish peroxidase-conjugated secondary antibodies (Amersham Biosciences, Uppsala, Sweden) for 1 h, and bands were detected by enhanced chemiluminescence (Amersham, Bucks, UK). Densitometric values were normalized to GAPDH levels. Values (protein/GAPDH) in control group were set to 100%.

### 2.5. MTS Assay

MTS assay was applied to reveal cell growth as previously reported [15], by the CellTiter 96® AQueous One Solution Cell Proliferation Assay Kit (Promega, Madison, WI, USA) accordingly. In each group, cells were cultured for 12, 24, and 48 h. At harvesting, 20 *μ*l of CellTiter 96 AQueous One Solution reagent was added to each well in a total volume of 100 *μ*l of medium for 3 h. Absorbance was measured at 450 nm using an ELISA plate reader. The growth rate was calculated from the absorbance, and the readings at 0 h time points in each group were set to 100%.

### 2.6. Flow Cytometry Assay

Annexin-V-FITC Apoptosis Detection Kit (BIPEC, USA) was used to determine apoptotic cells and PI staining was used to reveal cell cycle stage [15]. Briefly, cells were resuspended with 400 *μ*l binding buffer, labeled with annexin-V-FITC for 15 min and with PI for another 5 min; then cells were analyzed by flow cytometry by a FACScan flow cytometer (BD Biosciences, Mountain View, CA, USA).

### 2.7. Xenograft Mouse Model

Six- to eight-week-old male nude mice were kept on a 12 h light-dark cycle with access to food and water ad libitum. DU145 cells transfected with siRNA fragments (si-IGHG1 or si-Ctrl) for two days were collected and nude mice were subcutaneously injected with these DU145 prostate cancer cell xenografts (1 × 10^6^ DU145 cells). IGHG1 antibody was daily subcutaneously injected at the right axillary region of nude mice for four weeks. 30 days later, the mice were sacrificed by ketamin injection (50 mg/kg) and the xenografts were dissected out. After the measurement of tumor volume, xenografts were frozen in liquid nitrogen and stored at −80°C until further processing.

### 2.8. Immunohistochemistry

To analyze clinically collected samples, immunohistochemistry was performed as previously reported [15]. Briefly, paraffin sections were treated with hydrogen peroxide and later antigen retrieval was applied in a microwave in 10 mM citrate buffer. Then sections were fixed with paraformaldehyde and then permeabilized, blocked, and incubated with anti-IGHG1 and c-Myc antibodies. Immunostaining was analyzed with Super Sensitive Non-Biotin Polymer HRP Detection System according to the manufacturer's instructions (BioGenex, San Ramon, Canada).

### 2.9. Statistical Analysis

All experiments were repeated at least three times, and the results are presented as the mean ± SEM. Analyses of significance were performed using Student's* t*-tests or one-way ANOVAs, followed by Bonferroni corrections.* P* < 0.05 was considered statistically significant.

## 3. Results

### 3.1. Inhibition of IGHG1 Suppresses Cell Growth of Prostate Cancer

**Figure 1:**
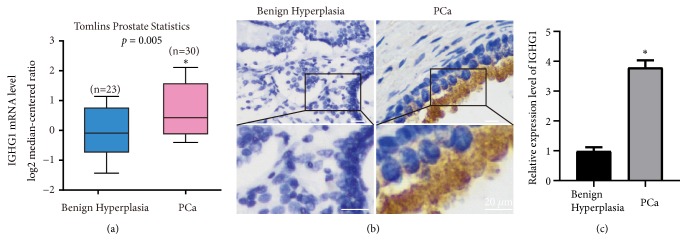
*The expression of IGHG1 in prostate cancer tissues.* (a) Database of Oncomine reveals the upregulation of IGHG1 in prostate cancers. (b) By immunohistochemistry detection, comparing to benign prostatic hyperplasia samples, IGHG1 is highly expressed in prostate cancer tissues. Scale bar, 20 *μ*m. (c) The statistical data of (b) were shown. *∗* donates* p* < 0.05.

In order to further determine the role of IGHG1, we firstly searched the Oncomine database for gene expressions in prostate cancer. As shown in Figure 1(a), the mRNA levels of IGHG1 were found significantly upregulated in prostate cancer samples compared to that in benign hyperplasia samples. Furthermore, we also collected prostate cancer and benign hyperplasia tissues clinically. And by immunohistochemical approach, we found that the protein expression of IGHG1 was also upregulated (Figures 1(b) and 1(c)).

**Figure 2:**
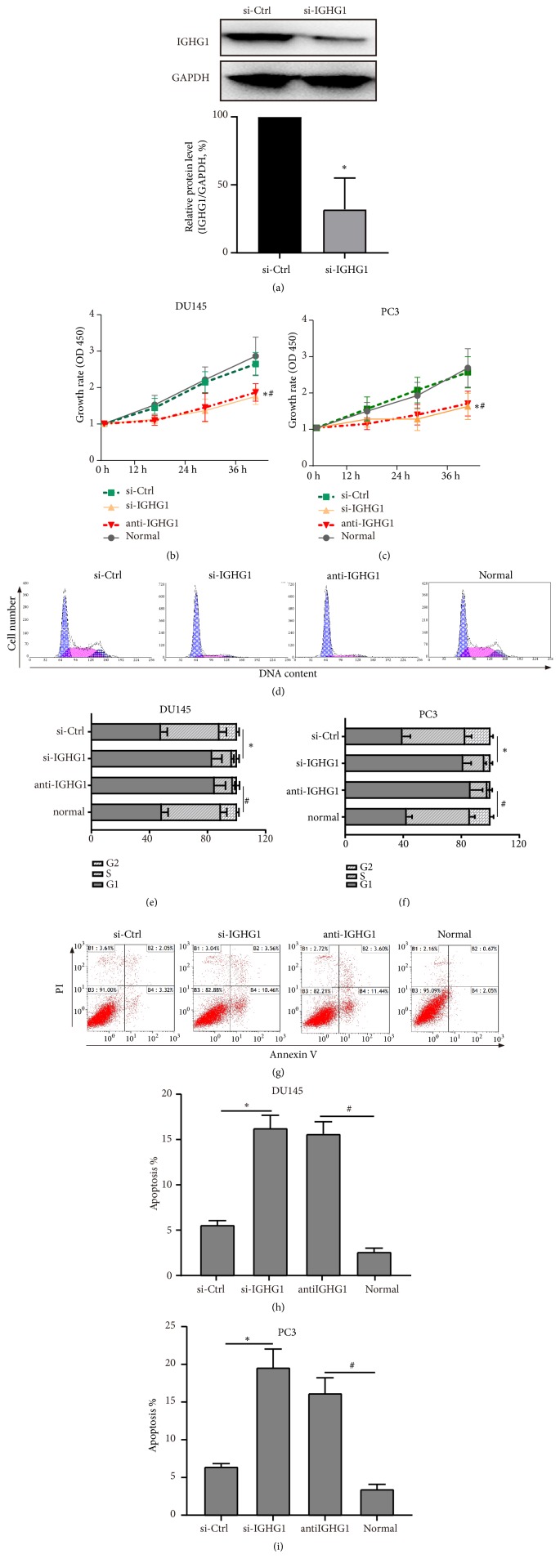
*Inhibition of IGHG1 suppresses the growth of PCa cells.* (a) Cultured DU145 cells were transfected with IGHG1 siRNA fragment, and the siRNA efficiency was confirmed by western blot. Then the DU145 (b) and PC3 (c) cells were subjected to MTS assay to evaluate the cell growth. The data of 0 h, 12 h, 24 h, and 48 h after transfection was shown as the growth rate. And the cells were stained with PI and Annexin-V to reveal cell cycle (d) and apoptosis (g) by flow cytometry technique. The statistical data were shown in (e), (h) in DU145 cells and (f), (i) in PC3 cells. All experiments were performed in triplicate, and results are expressed as the mean ± SD. *∗* denotes* p* < 0.05 versus si-Ctrl group; # denotes* p* < 0.05 versus normal control group.

**Figure 3:**
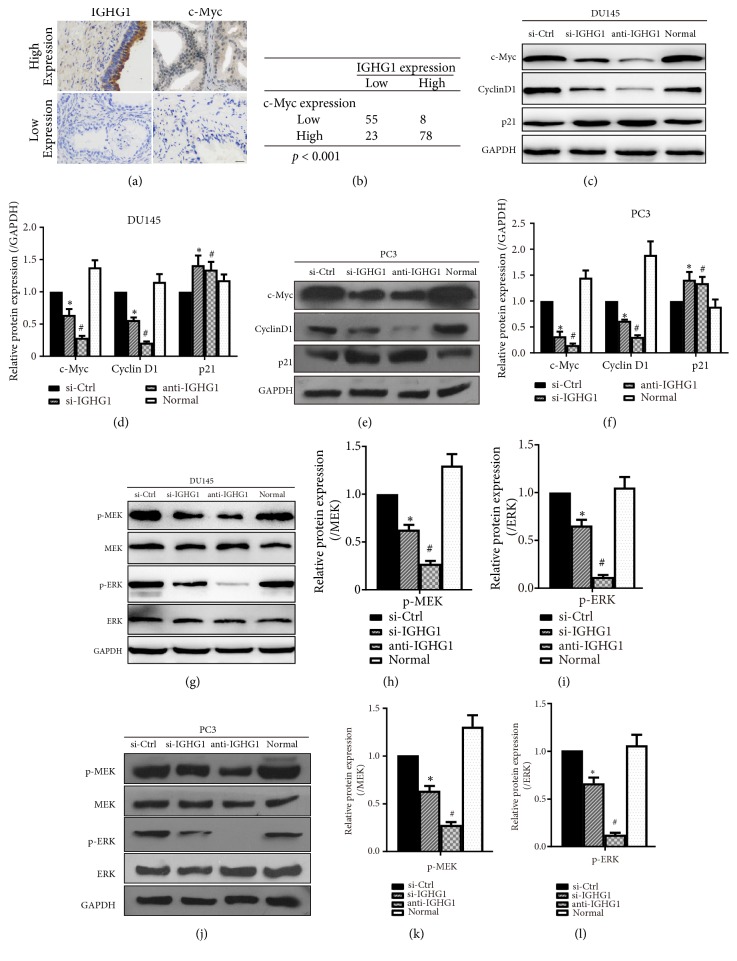
*IGHG1 regulates PCa cell growth via MEK/ERK/c-Myc pathway*. PCa tissue samples were subjected to immunohistochemistry; the representative images of IGHG1 and c-Myc expression were shown in (a) and the expression levels between the two proteins were shown in (b). DU145 cells (c, g) and PC3 cells (e, j) were transfected with IGHG1 siRNA fragment or si-Ctrl or with IGHG1 antibody and were subjected to western blot with c-Myc, Cyclin D1, p21, phosphor-MEK, total MEK, phosphor-ERK, and total ERK antibodies. GAPDH was used as loading control. The statistical data of expression of c-Myc, Cyclin D1, and p21 proteins (comparing to GAPDH) were shown in (d) and (f). The statistical data of p-MEK (comparing to total MEK, (h) and (k)) and p-ERK (comparing to total ERK, (i) and (l)) were shown as the mean ± SD. *∗* denotes* p* < 0.05 versus si-Ctrl group; # denotes* p* < 0.05 versus normal control group.

We previously designed siRNA fragments against IGHG1 and determined that genetic knockdown of IGHG1 suppressed prostate cancer cell growth [12]. Here, besides siRNA approach, we also applied IGHG1 antibody blocking to further identify the effect of IGHG1 on prostate cancer development. As shown in Figure 2(a), we determined the siRNA efficiency by western blot and found the working genetic knockdown affected the expression level of IGHG1 (si-IGHG1) in DU145 cells. By MTS assay, the cell growth rate of DU145 (Figure 3(b)) and PC3 (Figure 3(c)) cells was significantly inhibited by the transfection of siRNA fragments and by the addition of IGHG1 antibody (anti-IGHG1 group), comparing to control/normal culture groups. Furthermore, by flow cytometry with PI and Annexin-V staining, the cell cycle was arrested in si-IGHG1 and anti-IGHG1 groups (Figure 2(d)). The percentage of G1 phase was significantly upregulated under the inhibition of IGHG1 (Figures 2(e) and 2(f)). Inhibition of IGHG1 also induced cell apoptosis of prostate cancer cells (Figures 2(g)-2(h)). These data suggest that IGHG1 is important for the cell growth of prostate cancer, which is consistent with our previous results [12].

### 3.2. MEK/ERK/c-Myc Pathway Is Involved in IGHG1 Regulated PCa Cell Growth

Next, we explored the signaling pathway underlying IGHG1 regulated prostate cancer cell growth. By analyzing our immunohistochemical results from prostate cancer samples, we found that the expression of IGHG1 was positively correlated with c-Myc (Figure 3(a)). Among the 164 patients, 86 patients' samples were positive for IGHG1, whereas 78 patients' samples were negative for IGHG1. 101 patients' samples were positive for c-Myc, whereas 63 samples were negative for c-Myc (Figure 3(b)). These data suggested that IGHG1/c-Myc pathway was affected in patients with PCa. Thus, we determined whether the cell cycle proteins were involved in IGHG1 regulated PCa development. Levels of c-Myc, Cyclin D1, and p21 were evaluated by western blot, under the treatment of IGHG inhibition. The results showed that once IGHG1 was inhibited, the levels of c-Myc and Cyclin D1 were decreased, while the levels of p21 were upregulated (Figures 3(c) and 3(d) in DU145 cells; Figures 3(e) and 3(f) in PC3 cells).

**Figure 4:**
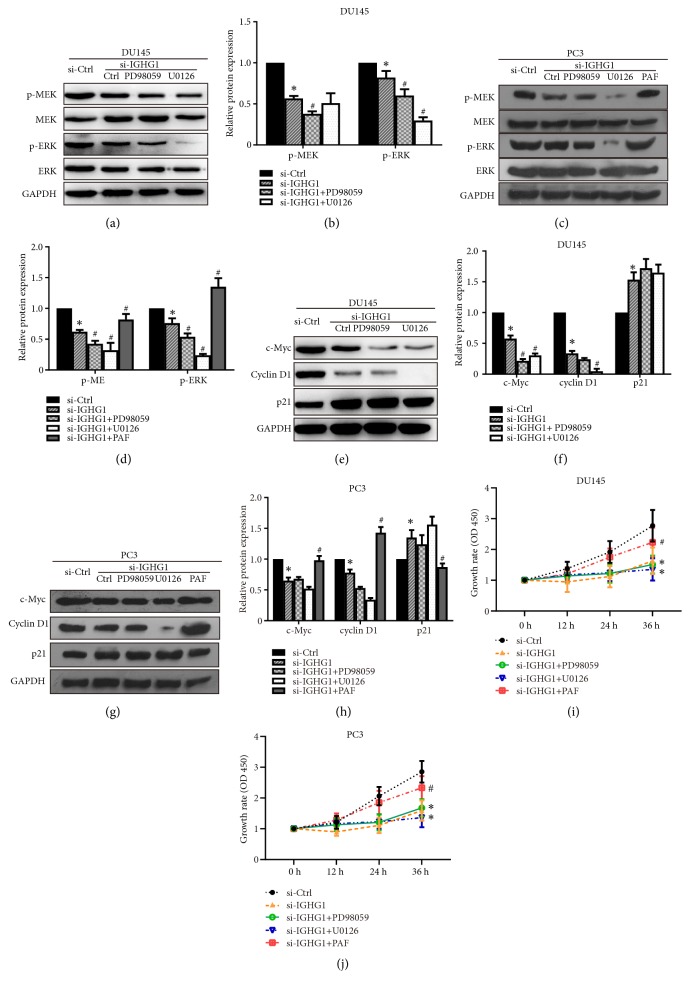
*Inhibition of MEK/ERK pathway by inhibitors confers the inhibition effect of IGHG1.* Cells transfected with or without IGHG1 siRNA fragments with added MEK/ERK inhibitors PD98059 and U0126 and MEK/ERK activator PAF(C-16). The cell lysates were subjected to western blot. The representative images were shown in (a), (e) in DU145 cells and (c), (g) in PC3 cells of activation of MEK and ERK and the statistical data were shown in (b), (f) in DU145 cells and (d), (h) in PC3 cells with the expression of c-Myc, Cyclin D1, and p21. The DU145 (i) and PC3 (j) cells with indicated administrations were subjected to MTS assay to evaluate the cell growth. The data of 0 h, 12 h, 24 h, and 48 h after transfection was shown as the growth rate. Data are expressed as the mean ± SD. *∗* denotes* p* < 0.05, compared with si-Ctrl group; # denotes* p* < 0.05, compared with si-IGHG1 group.

MAPKs/c-Myc axis has been reported in sustaining cancer development in many cancer types [16–18]. And the MEK/ERK/c-Myc pathway is involved in regulation of PCa cell growth [19]. Therefore, we determined the levels and activation of MEK/ERK under IGHG1 management. As shown in Figures 3(g)–3(l), the total levels of MEK and ERK remained unchanged upon the inhibition of IGHG1. However, the phosphorylation levels of MEK and ERK were significantly decreased when IGHG1 was inhibited, suggesting that the activation of MEK/ERK was inhibited. Statistical data were shown in Figures 3(h)–3(l) in both DU145 and PC3 cells. We further confirmed the data by pharmacological approaches, by using MEK/ERK inhibitors PD98059 and U0126. As shown in Figures 4(a)–4(d), inhibition of IGHG1 reduced the activation of MEK and ERK kinases and combination administration of PD98059 and U0126 further suppressed the levels of phosphorylated MEK and ERK levels. PAF(C-16) is an activator of MEK/ERK pathway, and in PC3 cells, the application of PAF(C-16) markedly reversed the inhibition effect of IGHG1 knockdown (Figures 4(c) and 4(d)). The statistical data were shown in Figures 4(b) and 4(d). Meanwhile, additional treatment with MEK/ERK inhibitors resulted in further decreased expression of c-Myc and Cyclin D1 and upregulated level of p21 (Figures 4(e)–4(h)). And activation of MEK/ERK pathway restored the levels of c-Myc and Cyclin D1 and suppressed p21 expressions (Figures 4(g) and 4(h)). Furthermore, inhibition of MER/ERK pathway was significantly suppressed while activation of that rescued the cell growth in both DU145 and PC3 cells upon IGHG1 genetic knockdown (Figures 4(i) and 4(j)). These data indicate that MEK/ERK/c-Myc pathway lies downstream of IGHG1 regulated prostate cancer cell growth.

### 3.3. Inhibition of IGHG1 Suppresses MEK/ERK/c-Myc Pathway* In Vivo*

**Figure 5:**
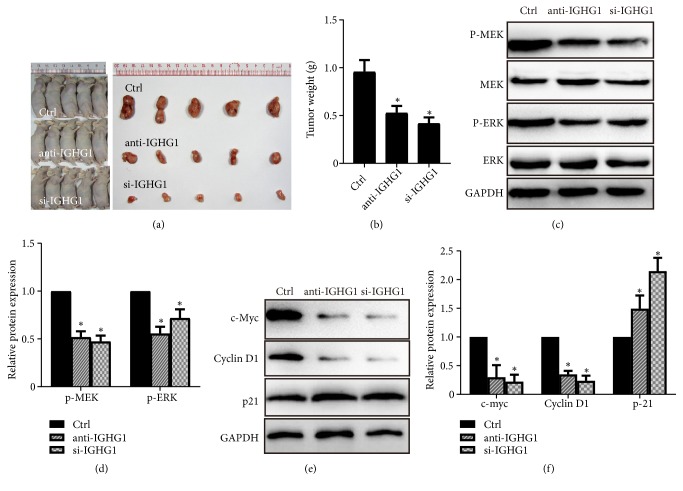
*IGHG1 functions via the MEK/ERK/c-Myc axis to regulate PCa tumor growth in vivo.* (a) DU145 cells were injected to nude mice, together with or without IGHG1 genetic silenced or IGHG1 antibody injection. One month later, mice were sacrificed, and the tumors were isolated. (b) The tumor weight was evaluated, and the samples lysates were subjected to western blot to detect the activation of MEK/ERK pathway ((c) and (d)) and the expression of c-Myc protein ((e) and (f)). Data are expressed as the mean ± SD. *∗* denotes* p* < 0.05.

To confirm the effect of IGHG1 on prostate cancer cell growth* in vivo*, we performed a xenograft assay of DU145 cell in athymic nude mice. Human prostate cancer DU145 cells were inoculated into nude mice subcutaneously, and IGHG1 antibody was simultaneously injected into the mice. Afterwards, the same amount of antibody was also injected every five days. DU145 cells with IGHG1 genetically silenced were injected as another group. One month later, mice bearing tumors were sacrificed and the tumors were isolated for further analysis. As shown in Figure 5(a), tumors in IGHG1 blocked group (anti-IGHG1 group) and IGHG1 silenced group (si-IGHG1) were much smaller than those in control mice. The tumor weight was decreased as shown in Figure 5(b). By western blot analysis, the expression levels of total MEK and ERK remained unchanged. However, the phosphorylated levels of MEK and ERK were significantly reduced by IGHG1 inhibition (Figures 5(c) and 5(d)). The expression levels of c-Myc and Cyclin D1 were decreased, while that of p21 was increased (Figures 5(e) and 5(f)). These data are consistent with the scenario* in vitro,* indicating that inhibition of IGHG1 inhibits the tumor growth of PCa via the MEK/ERK/c-Myc pathway* in vivo*.

## 4. Discussion

Prostate cancer is the third leading cause of cancer-related deaths of men in China. Endocrine therapy is an advanced treatment of prostate cancer; however, this approach would result in hormonal resistance [20, 21]. Moreover, once patients develop metastasis, the mortality rate of prostate cancer is extremely high. Thus, revealing the underlying mechanisms and identifying novel targets for treatment of prostate cancer have important significance. Here, in the current study, we found that IGHG1 was significantly upregulated in prostate cancer tissues. Inhibition of IGHG1 by genetic knockdown or antibody blocking markedly reduced the growth of prostate cancer cells and induced cell cycle arrest and cell apoptosis. Furthermore, MEK/ERK pathway was inactivated when IGHG1 was suppressed. Cell cycle related proteins, such as c-Myc, Cyclin D1, and p21, lied downstream of MEK/ERK pathway to mediate IGHG1 regulated prostate cancer.

The relationship between IGHG1 and many cancers has been identified during recent years. However, the role of IGHG1 in prostate cancer and the regulatory mechanisms remain largely unknown. We have revealed the presence of cytoplasmic and membranous IGHG1 in prostate cancer cell lines (LNCaP, PC3) previously and found that inhibition of IGHG1 by siRNA approach suppressed cell proliferation and induced apoptosis [12]. Here, we further supplemented our previous work and we showed that IGHG1 was upregulated in human prostate cancer tissues, comparing with benign hyperplasia samples (Figure 1). Administration of anti-IgG antibodies had been demonstrated to inhibit the growth of tumor cells in vitro and in mouse [5, 22]. Similar results were obtained by blocking of cancer-derived IgG to inhibit cancer cell growth [23]. Thus, using antibodies to block IGHG1 is an effective way besides siRNA approach, and we found that both of these methods resulted in similar effect* in vitro* and* in vivo*.

C-Myc plays critical role in PCa onset and progression; c-Myc overexpression induces neoplastic phenotype on human prostate normal epithelial cells [24], promotes PCa carcinogenesis during early stage [25, 26], confers androgen-independent growth [27], and induces tumor relapse after radiation therapy [28]. Furthermore, MAPK signaling, which is frequently deregulated in PCa [29], has been shown to promote the c-Myc gene expression [30]. Thus, dysregulated c-Myc oncoprotein expression is critical for PCa carcinogenesis [25, 31], leading to c-Myc being identified as a strategic therapeutic target for PCa treatment [32]. Inhibition of c-Myc transcription or reducing c-Myc stability and function has consequently used to counteract c-Myc protein accumulation in many cancers including PCa. Here, in our study, we found that c-Myc was overexpressed in PCa tissue samples, and c-Myc expression was positively correlated with IGHG1. Inhibition of IGHG1 by siRNA approach or by antibody blocking induced the downregulation of c-Myc, indicating that c-Myc lies downstream of IGHG1.

A variety of signaling pathways have been reported in the development of PCa and MAPKs have been reported to be closely related with PCa [29]. Here, the role played by MEK/ERK signaling in c-Myc protein was confirmed by means of selective and specific MEK/ERK inhibitor U0126 and PD98059. Firstly, increased activation of MEK and ERK was found with increased phosphorylation in cultured PCa cell line, and inhibition of IGHG1 reduced this activation, indicating that IGHG1 functioned via MEK/ERK/c-Myc axis to regulate PCa cancer growth. Moreover, MEK/ERK inhibitors significantly reduced phosphor-active MEK and ERK and downregulated c-Myc protein in DU145 cells. The inhibitors further enhanced the inhibition effect of IGHG1 suppression. Finally, by xenograft assay in nude mice, we confirmed that the inhibition of IGHG1 induced the downregulation of phosphor-activated MEK/ERK and expression of c-Myc, leading to the suppressed PCa growth* in vivo*. MEK/ERK/c-Myc axis may play a role in mediating response to radiation,* in vitro *and* in vivo* [19]. And radiation therapy induces the selection of aggressive PCa cells in an ERK-dependent manner [33]. We suspect that this may be due to the upregulated c-Myc expression via MEK/ERK activation.

In conclusion, the data in the current study further confirmed the role of IGHG1 in prostate cancer development. Inhibition of IGHG1 suppresses cancer cell growth* in vitro* and* in vivo*. Furthermore, IGHG1 functions via the MEK/ERK/c-Myc pathway and regulates the downstream cell cycle related proteins. These data elucidate the mechanisms of IGHG1 regulated prostate cancer cell proliferation, providing theoretical foundation and potential target for the clinical treatment of prostate cancer.

## Data Availability

The data used to support the findings of this study are available from the corresponding author upon request.
